# CHURN: TRANSITIONS OUT OF HEALTH SECTOR EMPLOYMENT FOR LOW-PREPARATION WORKERS, 2017–2022

**DOI:** 10.1093/geroni/igae098.0227

**Published:** 2024-12-31

**Authors:** Christine Bishop, Karen Donelan

**Affiliations:** Brandeis University, Waltham, Massachusetts, United States; Brandeis University, Waltham, Massachusetts, United States

## Abstract

Shortages and turnover for front-line paid caregivers and support staff in long-term services and supports for older adults undermine quality of care. These workers are part of the health sector workforce, and also participate in a larger labor market of jobs requiring minimal formal education and brief training. The Brandeis Front-line Health Worker Survey queried a random sample of U.S. residents screened for holding patient-facing jobs in the health sector between the survey date (Summer 2022) and five years prior (2017), generating information on a retrospective longitudinal cross section of US health sector workers. Respondents reporting occupations in Bureau of Labor Statistics Job Zones 1 and 2 were identified as participating in low-preparation labor markets. About half of low-preparation health sector workers reported work in nursing homes, residential settings, or home care. We estimated annual rates of transition into and out of heath sector employment for all low-preparation health workers for years prior to and during the pandemic. Descriptive logistic regressions showed the relationship of job churn to gender, caregiver status, and previous employment pattern. Despite concerns about health workforce stability as the pandemic wound down, low-preparation workers exhibited about the same year-to-year transition rates out of (and back into) the health sector throughout the period. This suggests that the Great Resignation in healthcare is a continuing pattern rather than a pandemic phenomenon. Employers and public policy makers setting wages and working conditions should recognize that front-line workers have, and have always had, alternatives beyond the health sector.

